# Supplementary material for: Mechanisms governing the pioneering and redistribution capabilities of the non-classical pioneer PU.1
Source: Nat Commun. 2020 Jan 21;11:402. doi: 10.1038/s41467-019-13960-2 (PMC6972792; doi:10.1038/s41467-019-13960-2)
Supplement: Supplementary file 7 — Source data [file 41467_2019_13960_MOESM7_ESM.zip › Source_Data/FigureS5/FigureS5B_MotifsETS1specificPeaks/homerResults/motif9.similar.html]

motif9

## Information for motif9

T
A
G
C
T
A
C
G
C
T
A
G
T
G
C
A
T
A
G
C
A
C
G
T
T
G
C
A
A
T
G
C
  
Reverse Opposite:  

T
A
C
G
A
C
G
T
T
G
C
A
A
T
C
G
A
C
G
T
G
A
T
C
A
T
G
C
A
T
C
G
  

|  |  |
| --- | --- |
| p-value: | 1e-53 |
| log p-value: | -1.237e+02 |
| Information Content per bp: | 1.597 |
| Number of Target Sequences with motif | 367.0 |
| Percentage of Target Sequences with motif | 32.33% |
| Number of Background Sequences with motif | 1056.1 |
| Percentage of Background Sequences with motif | 12.94% |
| Average Position of motif in Targets | 96.8 +/- 54.8bp |
| Average Position of motif in Background | 99.7 +/- 74.6bp |
| Strand Bias (log2 ratio + to - strand density) | 0.2 |
| Multiplicity (# of sites on avg that occur together) | 1.14 |
| Motif File: | file (matrix) reverse opposite |

### Similar de novo motifs found

|  |  |  |  |  |  |  |  |
| --- | --- | --- | --- | --- | --- | --- | --- |
| Rank | Match Score | Redundant Motif | P-value | log P-value | % of Targets | % of Background | Motif file |
| 1 | 0.921 | A C T G A C G T C G T A A C T G A C G T A G T C A G T C A C T G A G T C | 1e-31 | -71.947829 | 5.11% | 0.38% | motif file (matrix) |
| 2 | 0.796 | A G T C A C T G C G T A C G T A A G T C A C G T C G T A | 1e-14 | -34.062380 | 6.43% | 1.92% | motif file (matrix) |
